# Lipid profile of regular kratom (*Mitragyna speciosa* Korth.) users in the community setting

**DOI:** 10.1371/journal.pone.0234639

**Published:** 2020-06-11

**Authors:** Mohammad Farris Iman Leong Bin Abdullah, Kok Leng Tan, Salbiah Mohd Isa, Nur Sabrina Yusoff, Nelson Jeng Yeou Chear, Darshan Singh

**Affiliations:** 1 Lifestyle Science Cluster, Advanced Medical and Dental Institute, Universiti Sains Malaysia, Kepala Batas, Pulau Pinang, Malaysia; 2 Regenerative Medicine Cluster, Advanced Medical and Dental Institute, Universiti Sains Malaysia, Kepala Batas, Pulau Pinang, Malaysia; 3 Center for Drug Research, Universiti Sains Malaysia, Minden, Pulau Pinang, Malaysia; Chiang Mai University Faculty of Medicine, THAILAND

## Abstract

**Background and aim:**

Kratom, or *Mitragyna speciosa* Korth., is a tropical plant that has been reported to exhibit opioid-like effects. Although opioids have been demonstrated to alter the lipid profile of regular users, data on the lipid-altering effects of kratom are scarce. This study aimed to compare the fasting lipid profile of regular kratom users to that of healthy subjects who do not use kratom. It also determined the association between various characteristics of kratom users and the serum triglycerides, total cholesterol, low-density lipoprotein (LDL), and high-density lipoprotein (HDL) levels of regular kratom users.

**Methods:**

A total of 200 participants (n = 100 kratom users and n = 100 healthy subjects who do not use kratom) were recruited for this analytical cross-sectional study. Data on sociodemographic status, kratom use characteristics, cigarette smoking, physical activity, body mass index (BMI), fasting serum lipid profile, and liver function were collected from all participants.

**Results:**

The liver parameters of the study participants were within normal range. The serum total cholesterol and LDL of kratom users were significantly lower than those of healthy subjects who do not use kratom. There were no significant differences in the serum triglyceride and HDL levels. However, higher average daily frequency of kratom use and increasing age were associated with increased serum total cholesterol among kratom users. Other kratom use characteristics such as age of first kratom intake, duration of kratom use, and quantity of daily kratom intake were not associated with increased serum triglyceride, total cholesterol, LDL, and HDL levels.

**Conclusions:**

Our findings suggest regular kratom consumption was not linked to elevated serum lipids, except when there is a higher frequency of daily kratom intake. However, the study was limited by the small sample size, and hence a more comprehensive study with larger sample size is warranted to confirm the findings.

## Introduction

Kratom (*Mitragyna speciosa* Korth.) is a tropical plant, which can be found ubiquitously in Southeast Asia, such as in Malaysia, Thailand, and Indonesia [1]. Historically, rural inhabitants had been using kratom as a prophylactic to cure common health problems (e.g., pain, diarrhea, cough, etc.), as a stimulant drug for increasing work productivity, and as an opiate substitute [2,3]. Matured leaves are freshly harvested and eaten raw, or brewed and consumed as an herbal solution (e.g., kratom juice) [3]. Due to its pain mitigating effects [4], kratom use is increasing among opioid users, who use it as an alternative to prescription and illicit opioids in the United States to treat pain, opioid withdrawal, and psychological problems [5–7].

There are various alkaloids in kratom. Two of the most widely studied alkaloids with psychoactive properties and opioid-like effects include mitragynine and 7-hydroxymitragyine [4,8]. Despite the widespread sales of kratom products on the internet and convenience stores in the United States, and being backed by positive customer reviews regarding kratom’s pain-relieving effects [5–7], hitherto the US Food and Drug Administration (FDA) has not approve kratom use, as the herbal plant is alleged to have various risks, such as addiction, abuse, and dependence [9]. As a result of the incremental kratom toxicity recurrence [10,11], both the FDA and the Drug Enforcement Administration (DEA) are contemplating to regulate mitragynine and 7-hydroxymitragynine into Schedule 1 of the Control Substances Act [4].

At present, kratom’s safety profile remains poorly investigated. It is reported that kratom use is linked with a plethora of unpleasant health effects [12]. Commonly reported health problems associated with kratom use include cardiovascular problems (e.g., cardiomegaly, coronary atherosclerosis, hypertensive cardiovascular disease, and left ventricular hypertrophy) [12]. It remains unknown whether these reported health problems are direct consequences of kratom use per se or if they are compounded by other factors, such as underlying health issues or toxic effects rising from kratom poly-drug use [12]. So far, there are no studies to show that long-term kratom use can alter lipid profile and cause users to experience unpleasant health or cardiovascular risk. Given its opioid-like effects and the lack of data on its safety profile, further studies are needed to establish kratom’s purported therapeutic properties. Opioids have been shown to cause dyslipidemia in regular opioid users. In fact, several studies have shown that opioids have the potential to alter serum triglyceride and LDL levels and deplete HDL levels among opioid users [13,14].

Singh et al. (2018) reported significant differences in the lipid profiles of regular kratom users compared to healthy controls of people who do not use kratom. However, the study had a small sample size (n[kratom] = 58, n[control] = 19), did not measure the body mass index (BMI) of participants, and the random lipid profile was obtained instead of the fasting lipid profile [15]. Since kratom use has been implicated with several adverse health and toxic incidents [12], our study aimed to (a) determine the fasting lipid profile of regular kratom users and healthy subjects who don’t use kratom and (b) evaluate the associations between kratom use characteristics and serum triglyceride, serum total cholesterol, serum LDL, and serum HDL levels among kratom users.

## Materials and methods

### Study design and participant recruitment

**Fig 1:**
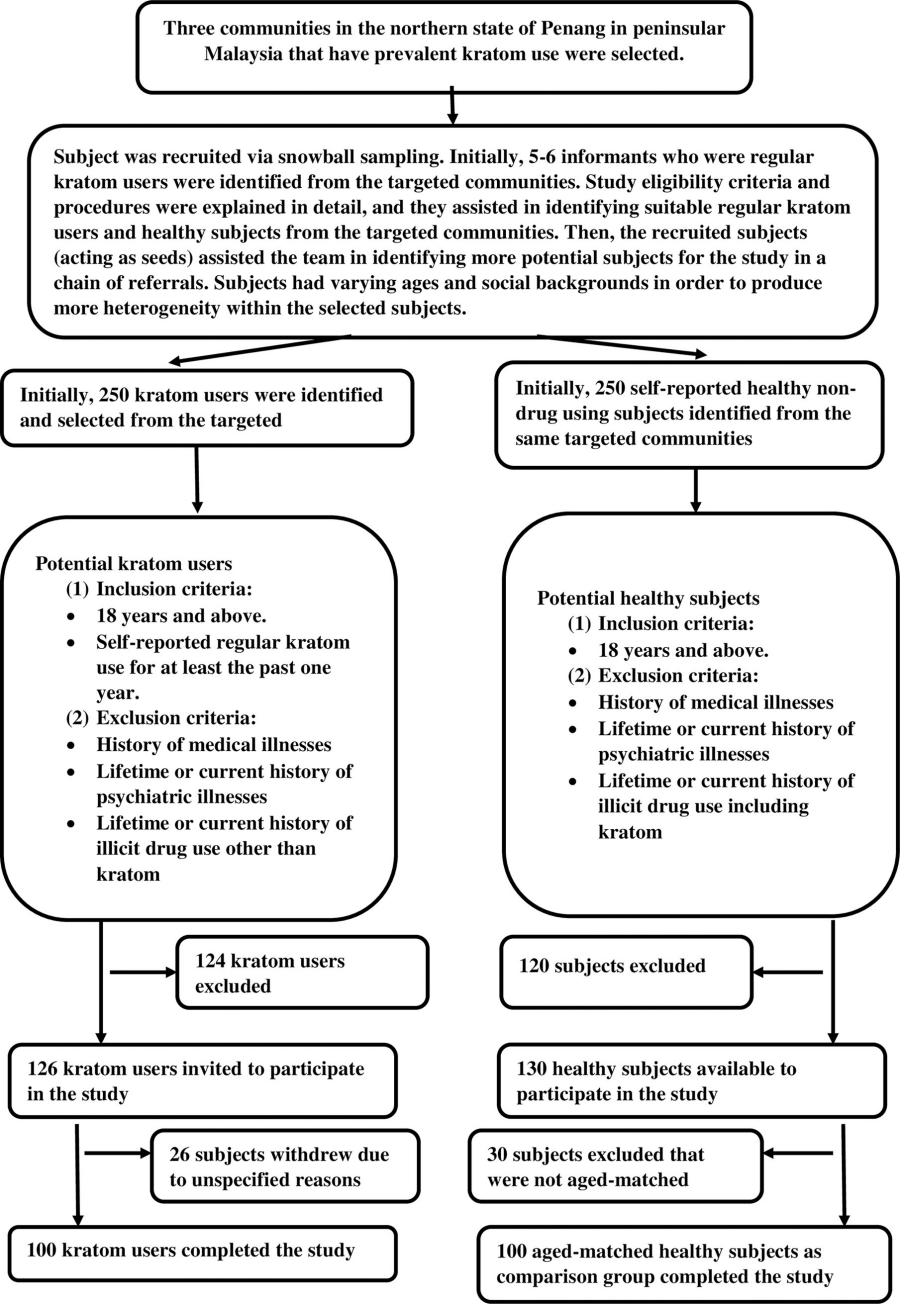
Sampling method and subject recruitment.

This analytical cross-sectional study was conducted over a duration of 9 months between May 1, 2019 and January 31, 2020 following approval obtained from the Human Ethics Committee of Universiti Sains Malaysia (code: USM/JEPeM/19010054). The sample size was calculated based on the formula for estimating the sample size for a multiple linear regression model (i.e., n = *50 + 8m*, where m is the number of independent variables) [16]. There were four multiple linear regression models in this study: (a) a model with serum triglycerides as the dependent variable and had seven predictors and hence required a sample size of 106 kratom users; a model with serum total cholesterol as the dependent variable and had six predictors and therefore required 98 kratom users; and two models with serum LDL and serum HDL as the dependent variables and had five predictors and hence required 90 kratom users. Therefore, the model with the largest estimated sample size required was the model with serum triglyceride as the dependent variable, which required 106 kratom users. We recruited kratom users and healthy subjects that didn’t use kratom from three communities that contained “hotspots” (locations where kratom users frequently visit to get their supply of kratom juice) via snowball sampling. This was done because of the difficulty to recruit kratom users since kratom has been considered a controlled poison in Malaysia since 2003 under the Dangerous Poisons Act 1952. The possession and smuggling of kratom leaves are considered offenses. Initially, 5–6 informants who were regular kratom users were identified from the targeted communities. They received a detailed explanation regarding the study eligibility criteria and procedures, and they assisted in identifying suitable regular kratom users and healthy subjects from the targeted communities. Then, the recruited subjects (acting as seeds) assisted the team with identifying more potential subjects for the study through a chain of referrals. Subjects of varying ages and demographics (marital status, education, employment status, monthly income, family support, and social circle) were chosen in order to produce more heterogeneity within the selected subjects. Subsequently, 250 kratom users and 250 healthy subjects who do not use kratom were identified and selected; after screening for eligibility criteria, 126 kratom users and 130 healthy subjects were invited to participate in the study. Then, 26 kratom users withdrew without giving specific reasons and 30 healthy subjects were excluded since they were not aged-matched with kratom users. Finally, 100 kratom users and 100 healthy subjects were recruited and completed the study. Although we attempted to enhance the heterogeneity of the recruited subjects, the selected sample size may not be truly representative of the entire kratom user population in the targeted communities. The sampling and subject recruitment methods are illustrated in Fig 1.

#### Inclusion and exclusion criteria

The inclusion criteria for kratom users were the following: (a) regular and daily kratom use for at least the past 1 year and (b) 18 years of age or older. The exclusion criteria were the following: (a) past or current history of using illicit drugs other than kratom, (b) history of lifetime or current psychiatric illnesses (such as psychotic disorders, depressive disorders, bipolar mood disorder, anxiety disorders, attention deficit hyperactivity disorder, and substance use and substance-related disorders), and (c) history of medical illnesses. The same inclusion and exclusion criteria were also applied to the healthy subjects, except that they must not have a previous or current history of illicit drug use including kratom use. Those who were interested in participating were given a detailed explanation of the study by the research team and were screened for eligibility; they signed an informed consent form prior to enrollment in the study.

#### Screening for medical illnesses

Screening for medical illnesses was carried out by a qualified physician in the research team to ensure that all the subjects selected for the study were free from any medical illnesses. The procedures performed were as follows: (1) Eliciting clinical history of all subjects, including regular intake of medications for treatment of any medical illnesses; (2) physical examination which included general examination, respiratory system, cardiovascular system, gastrointestinal tract, central and peripheral nervous system, genitourinary tract, and joints; and (3) blood investigations, including screening of full blood count, blood urea, creatinine and electrolytes, liver function test, fasting blood glucose, and thyroid function test. In all subjects selected for the study, we found no significant findings in the clinical history and physical examination. All blood tests during eligibility criteria screening of the selected subjects were within normal range.

#### Screening for psychiatric illnesses

Screening for psychiatric illnesses was performed by a qualified psychiatrist in the research team using the Diagnostic and Statistical Manual for Mental Disorders, Fifth Edition (DSM-V) diagnostic criteria [17] and the Mini International Neuropsychiatric Interview [18]. Those with a lifetime or current history of psychiatric illnesses−such as psychotic disorders, depressive disorders, bipolar mood disorder, anxiety disorders, attention deficit hyperactivity disorder, obsessive compulsive disorder, posttraumatic stress disorder, substance use, and substance-related disorders−were excluded.

#### Screening for lifetime and current history of illicit drug use

Comprehensive screening for lifetime and current history of illicit drug use was performed by a qualified psychiatrist on the research team. All potential subjects were screened for any history of opioids, amphetamine-type stimulant, cannabis, benzodiazepine, ketamine, phencyclidine, hallucinogens, inhalants, and cocaine use. Then, additional history regarding lifetime and current illicit drug use was also elicited from close family members and close contacts of the potential subjects. Finally, potential subjects were screened for opiate, amphetamine, methamphetamine, cannabis, methadone, buprenorphine, ketamine, PCP, and benzodiazepine using rapid urine-test-kits (Fast Screen Multi Drugs Dipcard, Reszon Diagnostics International Sdn. Bhd., Subang Jaya, Malaysia). Kratom users with illicit drug use other than kratom and healthy subjects with any illicit drug use including kratom were excluded.

### Data collection

Data collection was conducted in the community hall of the targeted communities. A semi-structured questionnaire was administered to participants by face-to-face interview to collect data on sociodemographic characteristics and clinical factors; these data included age (in years), gender (male/female), ethnicity (Malay/non-Malay), employment status (employed or self-employed/unemployed), cigarette smoker (non-smoker/1 to 5 cigarettes per day/6 to 10 cigarettes per day/more than 10 cigarettes per day), and weekly physical activity (inadequate physical activity/adequate physical activity). Physical activity is defined as any bodily movement due to skeletal muscles that required energy expenditure [19]. Moderate-intensity aerobic activities include playing tennis/badminton (doubles), brisk walking of at least 4 km, cycling at a speed of less than 16 km per hour, dancing, gardening, and water aerobics. Vigorous-intensity aerobic activities include running, cycling at a speed of at least 16 km per hour, playing tennis/badminton (singles), swimming in laps, hiking uphill, jumping rope, heavy house chores like continuous hoeing and digging, and aerobic dancing. Adequate physical activity is defined as moderate-intensity aerobic activities of at least 150 minutes per week or vigorous-intensity aerobic activities of at least 75 minutes per week [20]. Hence, in this study, physical activity was classified into two groups, i.e., (a) inadequate physical activity in which the participant engaged in moderate-intensity aerobic activities of < 150 minutes per week or vigorous-intensity aerobic activities of < 75 minutes per week and (b) adequate physical activity in which the participant engaged in moderate-intensity aerobic activities of ≥ 150 minutes per week or vigorous-intensity aerobic activities of ≥ 75 minutes per week. The BMI was also measured as weight (in kilogram) divided by the squared height (in metre^2^) of each participant. The median and interquartile range of the BMI of the participants was computed.

#### Kratom use characteristics of kratom users

Data on kratom use characteristics were also collected from regular kratom users, such as the first age of kratom use, duration of kratom use, the average frequency of daily kratom use, and the average quantity of daily kratom use. The first age of kratom use was investigated through the question, “What was your age when you started consuming kratom juice/tea?” The responses were coded as “before 18 years old” and “18 years old and above”. The duration of kratom use was recorded based on the question, “How many years have you been consuming kratom juice/tea?” The responses were coded as “1–5 years” and “more than 5 years.” Since all kratom users in this study consumed kratom on a daily basis, the average frequency of daily kratom use and the average quantity of daily kratom use were measured. The average frequency of daily kratom use was assessed with the question, “On average, how many times do you consume kratom juice/tea in a day?” Response categories ranged on a scale from 1 to 20 times. The average quantity of daily kratom consumption was measured through the question, “On average, how many glasses of kratom juice/tea do you consume in a day?” Response categories ranged on a scale from 1 to 30 glasses. The mean and standard deviation of the number of times per day and number of glasses per day of kratom juice/tea consumed respectively were computed. We measured the volume of kratom juice/tea consumed by the kratom users in a typical glass of kratom juice/tea in the communities which was at 350mL per glass.

#### Fasting lipid profile and liver function test

All participants were told to begin fasting at 9:00 p.m. and then arrive at the Advanced Medical and Dental Institute (AMDI), USM at 09:00 a.m. the following day for blood collection (i.e., 12 hours of fasting). A blood sample was collected from each participant for the fasting lipid profile (FLP) and liver function test (LFT). These tests were conducted in an ISO 15189-certified laboratory at AMDI, USM. Blood collection was performed by trained phlebotomists. Blood samples were transferred to the laboratory within one hour, and the FLP and LFT analyses were carried out on the same day. Quantitative determination of serum triglyceride (TG), total cholesterol (TC), HDL, total protein (TP), albumin, alkaline phosphatase (ALP), alanine transaminase (ALT), aspartate transaminase (AST), total bilirubin (TB) and direct bilirubin (DB) were performed on a fully automated Beckman Coulter AU680 biochemistry analyzer. Globulin was determined from simple subtraction of albumin from total protein value, and indirect bilirubin was determined by subtraction of direct bilirubin from total bilirubin value. LDL was calculated via Friedwald equation [21–23].

Total protein was quantified using the biuret method, which is suggested by the National Institutes of Standards and Technology (NIST) Standard Reference Material (SRM) 972c. Albumin was quantified using the bromocresol green method traceable to International Federation of Clinical Chemistry (IFCC) standard certified reference material (CRM) 470. ALP was quantified using the kinetic color (pNP) method traceable to Beckman Coulter master calibration, whereas ALT and AST both were measured using the kinetic UV method that is traceable to IFCC. Total bilirubin and direct bilirubin were quantified using the photometric color test (diazonium salt) that is traceable to Beckman Coulter master calibrator and NIST SRM 916a respectively. The results of TP, albumin, and globulin were expressed in g/L; ALP, ALT, and AST were expressed in U/L, and bilirubin was expressed in μmol/L.

Quantitation of TG, TC, and HDL levels were determined using the enzymatic color method. Quantitation of TG and TC is traceable to isotope dilution mass spectrometry and NIST SRM 909b Level 1 respectively. For HDL, the method is traceable to the US Centers for Disease Control and Prevention. All findings of FLP were expressed in mmol/L. Calibration for all test methods were accomplished by the use of chemistry calibrator cat. 66300, except HDL was determined with a separate HDL-cholesterol calibrator ODC 0011. Internal quality control procedures were implemented immediately following calibration in accordance to good laboratory practice. The lab protocol is available at dx.doi.org/10.17504/protocols.io.bfuwjnxe.

### Statistical analysis

All data analyses were performed with Statistical Package for Social Sciences (SPSS) version 24 (SPSS Inc., Chicago, IL, USA). Descriptive statistics for sociodemographic characteristics, clinical factors, kratom use characteristics, fasting lipid profile, and liver function test results were computed. The test for normality (with Shapiro-Wilk test and normal Q-Q plot) indicated the continuous variables were not normally distributed, except for liver function test variables. Hence, categorical variables were reported in frequencies and percentages, whereas continuous variables were reported as median and interquartile range (IQR), except for liver function test variables. Liver function test variables were described in mean and standard deviation. There were no missing data. Differences in the sociodemographic characteristics and clinical factors between kratom users and healthy subjects were evaluated using Pearson’s chi-square test or Fisher’s exact test for categorical variables and Mann-Whitney U test for continuous variables. Differences in liver function test variables between kratom users and healthy subjects were assessed using an independent t-test. To achieve the first objective of the study, differences in the fasting lipid profiles between kratom users and healthy subjects were evaluated with the Mann-Whitney U test. Then, the continuous variables were log transformed in order to be able to run the simple and multiple linear regression analyses. To achieve the second objective of the study, the individual associations between kratom use characteristics, such as the age of first kratom intake; duration of kratom use; average daily frequency of kratom use and average daily kratom consumption (independent variables); and serum triglyceride, serum total cholesterol, serum LDL, and serum HDL levels among kratom users (dependent variable), were assessed using simple linear regression analysis. We took into consideration possible confounding factors that may influence lipid profile, such as age, employment status, cigarette smoking, BMI, and physical activity [24–27]. Variables with p < 0.1 were entered into the multiple linear regression models as independent variables, and serum triglyceride, serum total cholesterol, serum LDL, and serum HDL levels were entered as dependent variables. To ascertain that the assumptions of the multiple linear regression models were met, we examined the following: (a) Multicollinearity was assessed by referring to the variance inflation factor (VIF), in which all the independent variables included in the multiple linear regression models had a score of < 5 [28]. (b) Homoscedasticity of the residuals were evaluated by examining the scatterplot of the standardized values of the regression residuals on the vertical axis against standardized predicted values on the horizontal axis. The residuals of all the models were roughly distributed in a rectangular shape in the scatterplot indicating homoscedasticity of the residuals [16,28]. (c) The normality of the residuals were examined by the Q-Q plot of the residuals of all the models which demonstrated all the points lied in a reasonably straight diagonal line from bottom left to top right, indicating no deviations from normality. Shapiro-Wilk test for normality also indicated that the residuals of all the models were normally distributed (p > 0.05) [16,28]. (d) The relationship between the independent variables and dependent variable in all the models could be modelled by a straight line in the scatterplot suggesting a linear relationship between the variables in all the models [16,28]. (e) Checking for outliers in all the models were performed by examining the scatterplot of the standardized values of the regression residuals on the vertical axis against standardized predicted values on the horizontal axis. None of the residuals in all the models lied more than three standardized units from the regression line [16,28]. Statistical significance was set at p < 0.05 for all analyses, and all p-values were two-sided.

## Results

### Participant characteristics

Table 1 lists the sociodemographic characteristics (age, gender, ethnicity, and employment) and clinical factors (cigarette smoking, BMI, and adequacy of physical activity) of all participants. A total of 200 participants were enrolled in this study (100 kratom users and 100 healthy subjects). All participants were males. There were no significant differences observed for sociodemographic characteristics, such as age, ethnicity, and employment status. Among the clinical factors assessed, significant differences were found for cigarette smoking and engagement in physical activity between kratom users and healthy subjects. There was no significant difference in the median BMI between kratom users and healthy controls, and the median BMI of the two groups were within normal range.

**Table 1:** Sociodemographic and clinical characteristics of the participants.

Variables	Kratom users		Healthy subjects		p-value
	N	%	N	%	
Age (years)	27^#^	19–43^$^	29^#^	26–34^$^	0.707^a^
Gender:					
Male	100	100	100	100	–
Ethnicity:					
Malay	99	99	95	95	
Non-Malay	1	1	5	5	0.212^b^
Employment status:					
Employed	86	86	89	89	
Unemployed	14	14	11	11	0.669^c^
Cigarette smoking:					
Non-smoker	0	0	62	62	
1–5 cigarettes/day	28	28	17	17	
6–10 cigarettes/day	14	14	14	14	
> 10 cigarettes/day	58	58	7	7	< 0.001^b^*
BMI (kg/m^2^)	22.51^#^	18.33–27.05 ^$^	22.91^#^	20.77–24.06^$^	0.436^a^
Physical activity:					
Inadequate physical activity	32	32	16	16	
Adequate physical activity	68	68	84	84	0.013^c^*

^#^ median

^$^ interquartile range

^a^ Mann-Whitney U test

^b^ Fisher’s exact test

^c^ Pearson’s chi-square test

* statistical significance at p < 0.05

Table 2 illustrates the kratom use characteristics of the regular kratom users in this study. Assessment of kratom use characteristics revealed that almost equal proportions of kratom users started using kratom at the age of < 18 years old (n = 48, 48%) and ≥ 18 years old (n = 52, 52%). A larger proportion of kratom users had been using kratom for more than 5 years (n = 56, 56%). The mean daily frequency of kratom use was 3.71 times/day (SD = 1.64), and the mean daily consumption of kratom was 4.49 glasses/day (SD = 1.99). The liver function test results of all participants (kratom users and healthy subjects) were within the normal range, and there were no differences in liver function parameters between the two groups (S1 Table).

**Table 2:** Kratom use characteristics of the study’s kratom users.

Variables	n =	%
Age of first kratom use:		
< 18 years old	48	48
≥ 18 years old	52	52
Duration of kratom use:		
1–5 years	44	44
> 5 years	56	56
Average daily frequency of kratom use:		
Mean number of times/day	3.71^#^	1.64^$^
Average daily consumption of kratom:		
Mean number of glasses/day	4.49^#^	1.99^$^

^#^ mean

^$^ standard deviation

### Fasting lipid profile of the participants

Table 3 shows the fasting lipid profiles of kratom users and healthy subjects. Of the lipid parameters tested, serum total cholesterol and serum LDL differed significantly between kratom users and healthy subjects. The serum LDL of kratom users was significantly lower than that of healthy subjects (median serum LDL [kratom] = 3.11 mmol/L, median serum LDL [healthy] = 3.62 mmol/L, p = 0.003). The serum total cholesterol of kratom users was also significantly lower than that of healthy subjects (median cholesterol [kratom] = 4.94 mmol/L, median cholesterol [healthy] = 5.56 mmol/L, p = 0.001). However, no significant differences in the serum triglyceride and serum HDL were detected between kratom users and healthy subjects.

**Table 3:** Fasting lipid profile of the participants.

Lipid variables	Kratom users		Healthy subjects		p-value
	Median	IQR	Median	IQR	
Serum triglycerides (mmol/L)	1.48	0.92–2.58	1.82	1.07–2.66	0.572
Serum total cholesterol (mmol/L)	4.94	4.35–6.10	5.56	4.95–6.19	0.001*
Serum LDL (mmol/L)	3.11	2.55–3.95	3.62	3.02–4.14	0.003*
Serum HDL (mmol/L)	1.24	1.07–1.39	1.2	1.07–1.36	0.598

* statistical significance at p < 0.05

### Association between kratom use characteristics and serum triglyceride level

Table 4 illustrates the associations between kratom use characteristics and serum triglyceride level among the kratom users, considering the confounding factors that may affect serum lipid profile, such as age, employment status, cigarette smoking, physical activity, and BMI. Simple linear regression revealed that kratom use characteristics, such as age of first kratom intake of > 18 years old and duration of kratom use for > 5 years, user age, unemployment, cigarette smoking of > 10 cigarettes/day, BMI, and adequate physical activity were significantly associated with serum triglyceride level (p < 0.1). Other kratom use characteristics, such as average daily frequency of kratom use and average daily consumption of kratom, were not associated with serum triglyceride level. However, none of the kratom use characteristics or other variables were significantly associated with serum triglyceride level when they were entered into a multiple linear regression model. The linear regression model contributed to a significant regression equation, i.e., F (7,92) = 6.929, p < 0.001, with an R^2^ of 0.345.

**Table 4:** Association between kratom use characteristics and serum triglyceride level among kratom users, accounting for confounding factors such as age, employment status, cigarette smoking, physical activity, and BMI.

Variables	Simple linear regression		p-value	Multiple linear regression model^a^		p-value
	B	95% CI		B	95% CI	
Age (years)	0.81	0.53–1.10	< 0.001*	0.29	-0.31–0.89	0.343
Employment status:						
Employed	Reference			Reference		
Unemployed	-0.18	-0.34–-0.03	0.023*	-0.30	-0.25–-0.05	0.174
Cigarette smoking:						
≤ 10 cigarettes/day	Reference			Reference		
> 10 cigarettes/day	0.12	0.003–0.23	0.045*	0.06	-0.04–0.16	0.239
BMI	1.42	0.91–1.93	< 0.001*	0.23	-0.70–1.16	0.623
Physical activity:						
Inadequate physical activity	Reference			Reference		
Adequate physical activity	-0.29	-0.40–-0.18	< 0.001*	-0.50	-0.32–0.04	0.126
Age of first kratom intake:						
< 18 years old	Reference			Reference		
≥ 18 years old	0.26	0.16–0.36	< 0.001*	0.07	-0.10–0.24	0.430
Duration of kratom use:						
1–5 years	Reference			Reference		
> 5 years	0.20	0.09–0.31	< 0.001*	0.002	-0.14–0.15	0.978
Average daily frequency of kratom use (mean number of times/day)	0.03	-0.01–0.06	0.113	-	-	-
Average daily consumption of kratom (mean number of glasses/day)	0.01	-0.02–0.03	0.679	-	-	-

* statistical significance at p < 0.1

^a^ multiple linear regression model reported F (7,92) = 6.929, p < 0.001, R^2^ = 0.345

### Association between kratom use characteristics and serum total cholesterol

Table 5 shows the associations between kratom use characteristics and serum total cholesterol level among the kratom users, considering the confounding factors that may affect serum lipid, such as of age, employment status, cigarette smoking, physical activity, and BMI. Simple linear regression indicated that kratom use characteristics, such as age of first kratom intake of > 18 years old, duration of kratom use for > 5 years, higher average daily frequency of kratom use, age, BMI, and adequate physical activity were significantly associated with serum total cholesterol level (p < 0.1). Average daily consumption of kratom was not associated with serum total cholesterol level. The multiple linear regression model revealed that higher average daily frequency of kratom use was significantly associated with an increase in serum total cholesterol. In addition, increasing age was also significantly associated with an increase in serum total cholesterol. Age of first kratom intake, duration of kratom use, and other variables entered in the model were not associated with the serum total cholesterol level. The linear regression model contributed to a significant regression equation of F (6,93) = 5.663, p < 0.001, with an R^2^ of 0.268.

**Table 5:** Association between kratom use characteristics and serum total cholesterol level among kratom users, accounting for confounding factors such as age, employment status, cigarette smoking, physical activity, and BMI.

Variables	Simple linear regression		p-value	Multiple linear regression model^a^		p-value
	B	95% CI		B	95% CI	
Age (years)	0.25	0.15–0.35	< 0.001*	0.25	0.04–0.47	0.022**
Employment status:						
Employed	Reference			-	-	-
Unemployed	-0.04	-0.10–0.01	0.135			
Cigarette smoking:						
≤ 10 cigarettes/day	Reference			-	-	-
> 10 cigarettes/day	0.02	- 0.01–0.06	0.213			
BMI	0.35	0.16–0.54	< 0.001*	0.32	-0.02–0.65	0.061
Physical activity:						
Inadequate physical activity	Reference			Reference		
Adequate physical activity	-0.05	-0.09–-0.01	0.010*	0.05	-0.02–0.11	0.159
Age of first kratom intake:						
< 18 years old	Reference			Reference		
≥ 18 years old	0.06	0.03–0.10	0.001*	-0.02	-0.08–0.04	0.518
Duration of kratom use:						
1–5 years	Reference			Reference		
> 5 years	0.06	0.03–0.10	0.001*	0.003	-0.05–0.05	0.899
Average daily frequency of kratom use (mean number of times/day)	0.01	0.005–0.02	0.059*	0.01	0.002–0.02	0.018**
Average daily consumption of kratom (mean number of glasses/day)	- 0.002	-0.01–0.01	0.667	-	-	-

* statistical significance at p < 0.1

** statistical significance at p < 0.05

^a^ multiple linear regression model reported F (6,93) = 5.663, p < 0.001, R^2^ = 0.268

### Association between kratom use characteristics and serum LDL

Table 6 shows the associations between kratom use characteristics and serum LDL level among the kratom users, accounting for the confounding factors that may affect serum lipid such as age, employment status, cigarette smoking, physical activity, and BMI. Initially, the simple linear regression revealed that age of first kratom intake of > 18 years old, duration of kratom use for > 5 years, as well as age, BMI, and adequate physical activity were significantly associated with serum LDL level (p < 0.1). Average daily frequency of kratom use and average daily consumption of kratom were not associated with serum LDL level. The multiple linear regression model indicated that higher BMI was significantly associated with increase in serum LDL among the kratom users. Other variables entered in the model were not associated with serum LDL level. The linear regression model contributed to a significant regression equation of F (5,94) = 4.965, p < 0.001 with an R^2^ of 0.209.

**Table 6:** Association between kratom use characteristics and serum LDL level among kratom users, accounting for confounding factors such as age, employment status, cigarette smoking, physical activity, and BMI.

Variables	Simple linear regression		p-value	Multiple linear regression model^a^		p-value
	B	95% CI		B	95% CI	
Age (years)	0.34	0.19–0.49	< 0.001*	0.26	-0.06–0.58	0.113
Employment status:						
Employed	Reference			-	-	-
Unemployed	-0.05	-0.13–0.30	0.215			
Cigarette smoking:						
≤ 10 cigarettes/day	Reference			-	-	-
> 10 cigarettes/day	0.04	- 0.02–0.10	0.161			
BMI	0.53	0.25–0.80	< 0.001*	0.50	0.02–1.00	0.050**
Physical activity:						
Inadequate physical activity	Reference			Reference		
Adequate physical activity	-0.08	-0.14–-0.02	0.009*	0.06	-0.04–0.16	0.233
Age of first kratom intake:						
< 18 years old	Reference			Reference		
≥ 18 years old	0.08	0.03–0.14	0.003*	-0.03	-0.12–0.06	0.561
Duration of kratom use:						
1–5 years	Reference			Reference		
> 5 years	0.09	0.04–0.15	0.001*	0.03	-0.05–0.10	0.453
Average daily frequency of kratom use (mean number of times/day)	0.01	-0.004–0.03	0.135	-	-	-
Average daily consumption of kratom (mean number of glasses/day)	-0.003	-0.02–0.01	0.672	-	-	-

* statistical significance at p < 0.1

** statistical significance at p < 0.05

^a^ multiple linear regression model reported F (5,94) = 4.965, p < 0.001, R^2^ = 0.209

### Association between kratom use characteristics and serum HDL

Table 7 shows the associations between kratom use characteristics and serum HDL level among the kratom users, accounting for confounding factors that may affect serum lipid, such as age, employment status, cigarette smoking, physical activity, and BMI. Initially, simple linear regression analysis indicated that kratom use characteristics, such as age of first kratom intake of > 18 years old and duration of kratom use for > 5 years, as well as age, BMI, and adequate physical activity were significantly associated with serum HDL level (p < 0.1). Average daily frequency of kratom use and average daily consumption of kratom were not associated with serum HDL level. However, the multiple linear regression model revealed that none of the variables entered in the model were associated with serum HDL level among the kratom users. The linear regression model contributed to a significant regression equation of F (5,94) = 3.453, p = 0.004, with an R^2^ of 0.182.

**Table 7:** Association between kratom use characteristics and serum HDL level among kratom users, accounting for confounding factors such as age, employment status, cigarette smoking, physical activity, and BMI.

Variables	Simple linear regression		p-value	Multiple linear regression model^a^		p-value
	B	95% CI		B	95% CI	
Age (years)	-0.09	-0.17–-0.004	< 0.039*	0.1	-0.08–0.27	0.268
Employment status:						
Employed	Reference			-	-	-
Unemployed	0.005	-0.04–0.05	0.821			
Cigarette smoking:						
≤ 10 cigarettes/day	Reference			-	-	-
> 10 cigarettes/day	- 0.02	-0.05–0.01	0.262			
BMI	-0.25	-0.39–-0.10	0.001*	-0.08	-0.35–0.19	0.562
Physical activity:						
Inadequate physical activity	Reference			Reference		
Adequate physical activity	0.06	0.03–0.09	< 0.001*	0.04	-0.02–0.09	0.167
Age of first kratom intake:						
< 18 years old	Reference			Reference		
≥ 18 years old	-0.03	- 0.06–-0.004	0.028*	-0.03	-0.08–0.20	0.251
Duration of kratom use:						
1–5 years	Reference			Reference		
> 5 years	-0.03	-0.06–-0.001	0.044*	-0.02	-0.06–0.02	0.282
Average daily frequency of kratom use (mean number of times/day)	0.01	-0.003–0.02	0.201	-	-	-
Average daily consumption of kratom (mean number of glasses/day)	-0.003	-0.01–0.005	0.439	-	-	-

* statistical significance at p < 0.1

^a^ multiple linear regression model reported F (5,94) = 3.453, p = 0.004, R^2^ = 0.182

## Discussion

This study determined the fasting lipid profiles of regular kratom users as compared to healthy subjects that do not use kratom. It evaluated the associations between kratom use characteristics and serum triglyceride, total cholesterol, LDL, and HDL levels, accounting for confounding factors, such as age, employment status, cigarette smoking, physical activity, and BMI among the kratom users [24–27]. We found that the serum total cholesterol and serum LDL levels were significantly lower in kratom users compared to healthy subjects who did not use kratom. There were no significant differences in the serum triglyceride and serum HDL levels between the kratom users and healthy subjects who did not use kratom. A Malaysian study that compared the blood parameters between regular kratom users and subjects who did not use kratom reported contrasting findings. Singh et al. (2018) found that the mean serum HDL was significantly higher in kratom users, whereas the mean serum cholesterol, LDL, and triglycerides did not differ significantly between users and non-users [15]. This discrepancy in findings may be due to differences in the methodology used in the study and differences in sample size. Additionally, as a comparison, the lipid profile findings in opioid users are inconsistent. In a study of opium users, Salman et al. (2010) reported significantly higher serum triglyceride, serum total cholesterol, and serum LDL levels in users compared to non-users [29], while another study in heroin addicts demonstrated significantly lower serum triglyceride and total cholesterol levels but higher serum VLDL when compared to non-addicts [30]. Another study of heroin addicts indicated that the drug users had significantly lower serum total cholesterol and HDL than the non-users, but no differences were seen in serum triglyceride levels [31]. The inconsistency of the lipid profile findings in opioid users may be due to the different routes of administration of the drug and the history of polysubstance use among the opioid addicts [32].

Unlike regular opioid use, our findings did not suggest elevation of serum lipid in regular kratom users, as it appears that kratom users had lower serum total cholesterol and LDL compared to healthy subjects who didn’t use kratom. It is unlikely that the lower serum total cholesterol and LDL levels were due to liver damage, which impairs biosynthesis of cholesterol, because the liver function test results of kratom users in our study were shown to be in the normal range. Although hepatotoxicity of kratom has been reported in a series of case reports in Western countries [33–37], it has not been reported in Southeast Asian nations such as Malaysia and Thailand [15,38]. This discrepancy may be a result of the co-ingestion of kratom with other substances, such as benzodiazepine, amphetamine, and ethanol, or kratom intake in those with underlying medical problems in the West. In contrast, in Southeast Asia, most of the kratom users only consumed kratom and are not poly-drug users [10,15,39]. The lower serum cholesterol and LDL levels in kratom users may not be due to physical activity since there was a significantly smaller proportion of kratom users who engaged in adequate physical activity, i.e., moderate-intensity physical activity of ≥ 150 minutes/week or vigorous-intensity physical activity of ≥ 75 minutes/week as compared to healthy subjects. It is also unlikely that lower serum cholesterol and LDL in kratom users is due to other confounding factors that may influence lipid profile; there were no differences in age and employment status between the kratom users and healthy subjects, and despite having higher proportion of regular smokers, kratom users still reported to have lower serum cholesterol and LDL.

However, evaluation of the associations between kratom use characteristics and the fasting lipid profiles of kratom users revealed that higher average daily frequency of kratom use was significantly associated with increased serum total cholesterol. We hypothesized that more frequent daily consumption of kratom may cause additive superposition that occurs due to multiple dosing of the same drug in short time intervals, where the total serum concentration of a drug is the sum of concentrations remaining from each prior dose combined with concentrations from the most recent dose. Hence, the increasing serum concentration of kratom alkaloids from multiple doses in a day leads to an increase in serum cholesterol [40]. This may be explained by the terminal half-life of the most abundant psychoactive alkaloid in kratom, i.e., mitragynine, which is 3–9 hours [41]. Hence, with multiple instances of kratom consumption on a daily basis, may lead to additive superposition in which the rate of mitragynine accumulation in the serum exceeded the rate of elimination of the alkaloid that the terminal half-life of mitragynine can compensate. Hence, the increase in the serum concentration of mitragynine may lead to high serum total cholesterol. Moreover, an animal study has shown that administration of morphine (mu opioid receptor agonist) for a duration of 5 to 28 days to mice increased serum triglycerides, LDL, and aortic cholesterol levels even in mice with a normal diet. These effects were prevented by administration of naltrexone (mu opioid receptor antagonist) [42,43]. These findings indicate that activation of mu opioid receptors in the central nervous system may lead to raised serum lipid levels. The psychoactive alkaloids of kratom, i.e., mitragynine and 7-hydroxymitragynine, are mu receptor agonists [1]; hence, its effect on serum cholesterol may be mediated through activation of mu receptors. However, the effect of mitragynine and 7-hydroxymitragynine on serum lipid has yet to be investigated. The other kratom use characteristics such as the age of first kratom intake, duration of kratom use, and average daily consumption of kratom were not predictive of any of the serum lipid parameters. In a study of regular kratom users in Malaysia, Singh et al. (2018) also found that the duration and quantity of daily kratom use were not associated with increased serum lipid levels [15]. In addition, increasing age and higher BMI among kratom users were also linked to increase serum cholesterol and LDL respectively. These findings are concordant with the findings in the general population that increasing age and BMI predict higher serum cholesterol and LDL [24,25].

The findings in this study must be interpreted in light of a few limitations. First, the sample size of this study was small, and the multiple linear regression model with serum triglycerides as the dependent variable should be interpreted with caution. Second, this study was a cross-sectional study; hence we were unable to establish the causal relationship between kratom use characteristics and serum lipid profile across time, and there is time-lag between kratom use and the lipid profiles of the kratom users. Third, all study participants were recruited from targeted communities in a single state in Malaysia, but kratom use is also common in other states in northern Peninsular Malaysia, such as Kedah and Perlis. Therefore, our findings cannot be generalized to represent the entire population in Malaysia that uses kratom. Fourth, the instructions for fasting were communicated verbally to the participants, but they were not admitted to the in-patient ward of the research facility to ensure that fasting occurred prior to blood collection. However, studies have shown that the differences in lipid parameters between fasting and non-fasting states are minimal and that the non-fasting lipid profile is not inferior to the fasting lipid profile as a predictive factor for cardiovascular diseases [44]. Finally, we were unable to recruit female kratom users in our study, as kratom use among females is not socially acceptable in Malaysia, so female users generally do not disclose their use. However, kratom use among females in the country is mainly in the form of traditional remedies on an as-needed basis for symptomatic relief of abdominal pain, muscle pain, diarrhea, and cough, rather than for regular use as seen in males [38].

Despite these limitations, we reported useful preliminary findings about the lipid profiles of regular kratom users without a history of poly-substance use, as compared to healthy subjects who didn’t use kratom and were from the same communities as the kratom users. However, further study of regular kratom users with a multicenter, prospective design (i.e., a cohort study) and inclusion of healthy subjects who don’t use drugs as a comparison group is recommended to confirm our findings.

## Conclusions

This study compared the fasting lipid profiles of regular kratom users with those of healthy subjects and evaluated the association between kratom use characteristics and various lipid parameters, which included serum triglyceride, cholesterol, LDL, and HDL among kratom users, accounting for confounding factors such as age, employment status, cigarette smoking, physical activity, and BMI. The serum cholesterol and LDL were significantly lower in the kratom users compared to the healthy subjects, which were independent of the effects of liver function, age, employment status, cigarette smoking, and physical activity. However, regardless of the amount of kratom consumed and duration of kratom use, higher average daily frequency of kratom use, increasing age, and higher BMI were associated with increased serum total cholesterol and LDL among kratom users. However, the findings in this study may have to be interpreted with caution due to the small sample size. Hence, further study using a larger sample size and improved methodology is warranted to confirm these findings.

## Supporting information

S1 Table Liver function test of the regular kratom users and healthy subjects. (DOCX)

S1 Appendix Socio-demographic, clinical factor and kratom use characteristics questionnaire use in this study. (DOCX)

## References

[1] Hassan Z, Muzaimi M, Navaratnam V, Yusoff NH, Suhaimi FW, Vadivelu R, et al From Kratom to mitragynine and its derivatives: physiological and behavioural effects related to use, abuse, and addiction. Neurosci Biobehav Rev. 2013;37(2):138–151. 10.1016/j.neubiorev.2012.11.012 23206666

[2] Suwanlert S. A study of kratom eaters in Thailand. Bull Narc. 1975;27(3):21–27. 1041694

[3] Vicknasingam B, Narayanan S, Beng T, Mansor SM. The informal use of ketum (*Mitragyna speciosa*) for opioid withdrawal in the northern states of peninsular Malaysia and implications for drug substitution therapy. Int J Drug Policy. 2010;21(4):283–288. 10.1016/j.drugpo.2009.12.003 20092998

[4] Prozialeck WC, Avery BA, Boyer EW, Grundmann O, Henningfield JE, Kruegel AC, et al Kratom policy: The challenge of balancing therapeutic potential with public safety. Int J Drug Policy. 2019;70:70–77. 10.1016/j.drugpo.2019.05.003 31103778 PMC7881941

[5] Grundmann O. Patterns of Kratom use and health impact in the US-Results from an online survey. Drug Alcohol Depend. 2017;176:63–70. 10.1016/j.drugalcdep.2017.03.007 28521200

[6] Garcia-Romeu A, Cox DJ, Smith KE, Dunn KE, Griffiths RR. Kratom (*Mitragyna speciosa*): User demographics, use patterns, and implications for the opioid epidemic. Drug Alcohol Depend. 2020 (Forthcoming). 10.1016/j.drugalcdep.2020.107849 PMC7423016 32029298

[7] Coe MA, Pillitteri JL, Sembower MA, Gerlach KK, Henningfield JE. Kratom as a substitute for opioids: Results from an online survey. Drug Alcohol Depend. 2019;202:24–32. 10.1016/j.drugalcdep.2019.05.005 31284119

[8] Kruegel AC, Gassaway MM, Kapoor A, Váradi A, Majumdar S, Filizola M, et al Synthetic and receptor signaling explorations of the mitragyna alkaloids: mitragynine as an atypical molecular framework for opioid receptor modulators. J Am Chem Soc. 2016;138(21):6754–6764. 10.1021/jacs.6b00360 27192616 PMC5189718

[9] U. S. Food and Drug Administration. FDA issues warnings to companies selling illegal, unapproved kratom drug products marketed for opioid cessation, pain treatment and other medical uses; 2019. [Cited 27 February 2020]. Available from: https://www.fda.gov/news-events/press-announcements/fda-issues-warnings-companies-selling-illegal-unapproved-kratom-drug-products-marketed-opioid.

[10] Anwar M, Law R, Schier J. Notes from the field: kratom (*Mitragyna speciosa*) exposures reported to poison centers—United States, 2010–2015. CDC. 2016;65(29):748–749. 10.15585/mmwr.mm6529a4 27466822

[11] Post S, Spiller HA, Chounthirath T, Smith GA. Kratom exposures reported to United States poison control centers: 2011–2017. Clin Toxicol. 2019;57(10):847–854. 10.1080/15563650.2019.1569236 30786220

[12] Corkery JM, Streete P, Claridge H, Goodair C, Papanti D, Orsolini L, et al Characteristics of deaths associated with kratom use. J Psychopharmacol. 2019;33(9):1102–1123. 10.1177/0269881119862530 31429622

[13] Rahimi N, Gozashti MH, Najafipour H, Shokoohi M, Marefati H. Potential effect of opium consumption on controlling diabetes and some cardiovascular risk factors in diabetic patients. Addict Health. 2014;6(1–2):1–6. 25140211 PMC4137437

[14] Aghadavoudi O, Eizadi-Mood N, Najarzadegan MR. Comparing cardiovascular factors in opium abusers and non-users candidate for coronary artery bypass graft surgery. Adv Biomed Res. 2015;4:12 Available from: 10.4103/2277-9175.148294 25625118 PMC4300596

[15] Singh D, Müller CP, Murugaiyah V, Hamid SBS, Vicknasingam BK, Avery B, et al Evaluating the hematological and clinical-chemistry parameters of kratom (*Mitragyna speciosa*) users in Malaysia. J Ethnopharmacol. 2018;214:197–206. 10.1016/j.jep.2017.12.017 29248450

[16] Tabachnick BG, Fidell LS. Using multivariate statistics. 3^rd^ ed New York: Harper Collins; 1996.

[17] American Psychiatric Association. Diagnostic and Statistical Manual of Mental Disorders (DSM-5). 5^th^ Ed Arlington: American Psychiatric Publishing; 2013.

[18] Sheehan DV, Lecrubier Y, Sheehan KH, Amorim P, Janavs J, Weiller E, et al The Mini-International Neuropsychiatric Interview (M.I.N.I.): the development and validation of a structured diagnostic psychiatric interview for DSM-IV and ICD-10. J Clin Psychiatry. 1998;59(Suppl 20:22–33):quiz 34–57. 9881538

[19] World Health Organization. Physical activity; 2018. [Cited 7 February 2020]. Available from: https://www.who.int/news-room/fact-sheets/detail/physical-activity.

[20] U.S. Department of Health and Human Services. Physical Activity Guidelines for Americans, 2nd edition. Washington, DC: U.S; 2018. [Cited 11 February 2020]. https://health.gov/sites/default/files/201909/Physical_Activity_Guidelines_2nd_edition.pdf.

[21] Albers JJ, Warwick GR, Cheng MC. Determination of high-density lipoprotein (HDL)-cholesterol. Lipids. 1978;13:926–932. 10.1007/BF02533852 220488

[22] Tietz NW. Clinical guide to laboratory tests. 2^nd^ ed Philadelphia: WB Saunders; 1990.

[23] Johnson R, McNutt P, MacMahon S, Robson R. Use of the Friedewald formula to estimate LDL-cholesterol in patients with chronic renal failure on dialysis. Clin Chem. 1997;43(11):2183–2184. 9365406

[24] Ni WQ, Liu XL, Zhuo ZP, Yuan XL, Song JP, Chi HS, et al Serum lipids and associated factors of dyslipidemia in the adult population in Shenzhen. Lipids Health Dis. 2015;14:71 Available from: 10.1186/s12944-015-0073-7 26168792 PMC4501051

[25] Agongo G, Nonterah EA, Debpuur C, Amenga-Etego L, Ali S, Oduro A, et al The burden of dyslipidaemia and factors associated with lipid levels among adults in rural northern Ghana: an AWI-Gen sub-study. PLoS ONE. 2019;14(2):e0213233 Available from: 10.1371/journal.pone.0213233 30811505 PMC6392312

[26] Szapary PO, Bloedon LT, Foster GD. Physical activity and its effects on lipids. Curr Cardiol Rep. 2003;5(6):488–493. 10.1007/s11886-003-0112-2 14558992

[27] Mitchell BD, Kalra G, Ryan KA, Zhang M, Sztalryd C, Steinle NI, et al Increased usual physical activity is associated with a blunting of the triglyceride response to a high-fat meal. J Clin Lipidol. 2019;13(1):109–114. 10.1016/j.jacl.2018.11.006 30553757 PMC6379118

[28] Aljandali A. Multivariate methods and forecasting with IBM® SPSS® statistics. Cham: Springer Nature; 2017.

[29] Salman TM, El Zahaby MM, Mansour OA, Omran GA, Gomma SM, Gad HS. Oxidative stress and lipotoxicity of bhang and opium addiction. Effects on adrenal gland secretions. Dyn Biochem Proc Biotechnol Mol Biol. 2010;4:50–54.

[30] Qasini A, Choudhry N, Mahmood S, Riaz I, ul Haq MF, Adnan N. Evaluation of lipids and lipoprotein levels in opium and heroin addicts in Punjabi population. Esculapio. 2013;9:163–167.

[31] Kouros D, Tahereh H, Mohammadreza A, Minoo MZ. Opium and heroin alter biochemical parameters of human's serum. Am J Drug Alcohol Abuse. 2010;36(3):135–139. 10.3109/00952991003734277 20465370

[32] Najafipour H, Beik A. The impact of opium consumption on blood glucose, serum lipids and blood pressure, and related mechanisms. Front Physiol. 2016;7:436 Available from: 10.3389/fphys.2016.00436 27790151 PMC5061814

[33] Kapp FG, Maurer HH, Auwarter V, Winkelmann M, Hermanns-Clausen M. Intrahepatic cholestasis following abuse of powdered kratom (*Mitragyna speciosa*). J Med Toxicol. 2011;7(3):227–231. 10.1007/s13181-011-0155-5 21528385 PMC3550198

[34] Dorman C, Wong M, Khan A. Cholestatic hepatitis from prolonged kratom use: a case report. Hepatology. 2015;61(3):1086–1087. 10.1002/hep.27612 25418457

[35] Tayabali K, Bolzon C, Foster P, Patel J, Kalim MO. Kratom: a dangerous player in the opioid crisis. J Community Hosp Intern Med Perspect. 2018;8(3):107–110. 10.1080/20009666.2018.1468693 29915645 PMC5998276

[36] Fernandes CT, Iqbal U, Tighe SP, Ahmed A. Kratom-induced cholestatic liver injury and its conservative management. J Investig Med High Impact Case Rep. 2019. (Forthcoming). 10.1177/2324709619836138 PMC6440031 30920318

[37] Osborne CS, Overstreet AN, Rockey DC, Schreiner AD. Drug-induced liver injury caused by kratom use as an alternative pain treatment amid an ongoing opioid epidemic. J Investig Med High Impact Case Rep. 2019;7:1–5. 10.1177/2324709619826167 PMC6350132 30791718

[38] Singh D, Narayanan S, Vicknasingam B. Traditional and non-traditional uses of Mitragynine (Kratom): A survey of the literature. Brain Res Bull. 2016;126(Pt 1):41–46. 10.1016/j.brainresbull.2016.05.004 27178014

[39] Kronstrand R, Roman M, Thelander G, Eriksson A. Unintentional fatal intoxications with mitragynine and O-desmethyltramadol from the herbal blend Krypton. J Anal Toxicol. 2011;35(4):242–247. 10.1093/anatox/35.4.242 21513619

[40] Bush MA. Repeated doses: key pharmacokinetic consideration. [Cited February 6 2020]. Available from: https://www.nuventra.com/resources/blog/repeated-doses/.

[41] Ya K, Tangamornsuksan W, Scholfield N, Methaneethorn J, Lohitnavy M. Pharmacokinetics of mitragynine, a major analgesic alkaloid in kratom (*Mitragyna speciosa*): A systematic review. Asian J Psychiatr. 2019;43:73–82. 10.1016/j.ajp.2019.05.016 31100603

[42] Bryant HU, Story JA, Yim GKW. Morphine-induced alterations in plasma and tissue cholesterol levels. Life Sci. 1987;41(5):545–554. 10.1016/0024-3205(87)90406-1 3600193

[43] Bryant HU, Story JA, Yim GKW. Stress and morphine-induced elevations of plasma and tissue cholesterol in mice: reversal by naltrexone. Biochem Pharmacol. 1988;37(19):3777–3781. 10.1016/0006-2952(88)90415-7 3178891

[44] Langsted A, Nordestgaard BG. Nonfasting versus fasting lipid profile for cardiovascular risk prediction. Pathology. 2019;51(2):131–141. 10.1016/j.pathol.2018.09.062 30522787

