# Supplementary material for: Lipid profile of regular kratom (Mitragyna speciosa Korth.) users in the community setting
Source: PLoS One. 2020 Jun 11;15(6):e0234639. doi: 10.1371/journal.pone.0234639 (PMC7289408; doi:10.1371/journal.pone.0234639)
Supplement: S1 Table — (DOCX) [file pone.0234639.s001.docx]

**S1 Table. Liver function test of the regular kratom users and healthy subjects**

| **Blood parameters** | **Kratom users** | **Healthy subjects** | **Reference ranges** | **p-value** |
| --- | --- | --- | --- | --- |
| Mean total protein (g/L) | 75.25 ± 3.38 | 76.63 ± 3.28 | 66 to 83 | 0.423 |
| Mean serum albumin (g/L) | 41.63 ± 1.06 | 43.13 ± 2.48 | 35 to 52 | 0.125 |
| Mean serum globulin (g/L) | 33.63 ± 3.20 | 31.75 ± 3.01 | 23 to 35 | 0.343 |
| Albumin/globulin ratio | 1.25 ± 0.12 | 1.44 ± 0.17 | 1.10 to 2.10 | 0.056 |
| Mean total bilirubin (µmol/L) | 9.75 ± 1.67 | 12.38 ± 1.46 | 5 to 21 | 0.082 |
| Mean alanine transaminase (ALT) (IU/L) | 19.25 ± 1.19 | 26.38 ± 3.45 | < 50 | 0.252 |
| Mean aspartate transaminase (AST) (IU/L) | 20.63 ± 2.17 | 29.50 ± 2.18 | < 50 | 0.093 |
| Mean alkaline phosphatase (ALP) (IU/L) | 90.88 ± 8.03 | 98.88 ± 5.83 | 30 to 120 | 0.504 |
