# Supplementary material for: Lipid profile of regular kratom (Mitragyna speciosa Korth.) users in the community setting
Source: PLoS One. 2020 Jun 11;15(6):e0234639. doi: 10.1371/journal.pone.0234639 (PMC7289408; doi:10.1371/journal.pone.0234639)
Supplement: S1 Appendix — (DOCX) [file pone.0234639.s002.docx]

**S1 Appendix. Socio-demographic, clinical factor and kratom use characteristics questionnaire use in this study**

**Details regarding the socio-demographic, clinical and kratom use characteristics questionnaire:**

The original Malay version of the socio-demographic, clinical and kratom use characteristics questionnaire was initially drafted by a team of kratom experts which comprised of 4 members i.e. a cardiologist, a chemical pathologist, a psychiatrist, and a drug addiction researcher, who are all proficient in the Malay language. Then, the sentence structure, wordings, semantic and language quality of the drafted questionnaire were checked by two of the content experts who native language is Malay. Then, the drafted questionnaire was pre-tested in a pilot study which involved 20 regular kratom users and 20 healthy subjects who were not included in the study to test for the comprehensiveness of their sentence structure, words and instructions, their semantic quality, and the appropriateness of the duration of administration of the questionnaire. After the subjects have answered the questions, we conducted a face-to-face interview in which the subjects were asked to point out any redundant words and sentence structure which they could not comprehend, any language errors and the appropriateness of the duration of administration. If there is any feedback regarding inappropriateness of the questionnaire, the feedback will be reviewed by the group of experts who will restructure the drafted Malay version before the final draft of the questionnaire is constructed. The procedure was in accordance with the standard procedures on how face and content validity was determined as recommended by the World Health Organization [1].

Our findings in the pilot study shown that three fourth of the participants agreed that the clarity, semantic quality and comprehensibility of the words, instructions and sentence structures in the Malay versions of the questionnaire as well as the duration of administration were appropriate. While one fourth of the participants even commented that the above parameters were strongly appropriate. None of the participants indicate any items in the Malay version of the socio-demographic, clinical and kratom use characteristics questionnaire were not appropriate. Hence, there was no need for restructuring of the questionnaire by the group of experts and the final draft was use for the study. The Malay version of the socio-demographic, clinical and kratom use characteristics questionnaire was not validated further.

**Reference:**

1. World Health Organization. Process of translation and adaptation of instruments. [Cited 25 March 2019]. Available from: <https://www.who.int/substance_abuse/research_tools/translation/en/>

**Part 1: Socio-demographic and clinical characteristics questionnaire (English version):**

**Research code:**

**Date:**

**Instruction: Below are the demographic and clinical questions to be answered by all the participants. Please answer all the questions accordingly.**

*** Please tick the appropriate box whether you are using kratom or not.**

I am using kratom I never use kratom

**(If you are using kratom please proceed to Part 2 after answering questions in Part 1)**

**Section A: Socio-demographic characteristics**

(1) What is your age?

years

(2) What is your gender?

Male Female

(3) What is your ethnicity?

Malay Non-Malay

(4) What is your employment status?

Employed or doing own business Unemployed

**Section B: Clinical characteristics**

(1) Do you smoke cigarettes?

I am a smoker I am a non-smoker

(a) If you are a smoker, what is the average number of cigarettes which you smoke in a day?

1-5 cigarettes per day

6-10 cigarettes per day

More than 10 cigarettes per day

(2) Are you engaging in adequate physical activity in a week? (Please take note that: Moderate-intensity aerobic activities include playing tennis/badminton (doubles), brisk walking of at least 4 km, cycling for less than 16 km per hour, dancing, gardening, water aerobics, etc. Vigorous-intensity aerobic activities include running, cycling for at least 16 km per hour, playing tennis/badminton (singles), swimming in laps, hiking uphill, jumping rope, heavy house chores like continuous hoeing and digging, aerobic dancing, etc.).

Adequate physical activity (I engaged in moderate-intensity aerobic activities of at

least 150 minutes per week or vigorous-intensity aerobic activities of at least 75

minutes per week)

Inadequate physical activity (I engaged in moderate-intensity aerobic activities of

less than 150 minutes per week or vigorous-intensity aerobic activities of less than

75 minutes per week

(3) Please take note that one of our staff will measure your weight and height to assess your body mass index (BMI).

Weight= kg

Height= m

BMI= kg/m^2^

**Part 2. Kratom use characteristics questionnaire (English version):**

**Instruction: Below are the kratom use characteristics questions to be answered by those who are using kratom. Please answer all the questions accordingly.**

(1) What was your age when you started consuming kratom juice/tea?

Before 18 years old

18 years old and above

(2) How many years have you been consuming kratom juice/tea?

1-5 years

More than 5 years

(3) On average, how many times do you consumed kratom juice/tea in a day? Please circle your answer.

1 time per day/2 times per day/3 times per day/4 times per day/5 times per day/6 times per day/7 times per day/8 times per day/9 times per day/10 times per day/11 times per day/12 times/day/13 times per day/14 times per day/15 times per day/16 times per day/17 times per day/18 times per day/19 times per day/20 times per day

(4) On average, how many glasses of kratom juice/tea do you consumed in a day? Please circle your answer.

1 glass per day/2 glasses per day/3 glasses per day/4 glasses per day/5 glasses per day/6 glasses per day/7 glasses per day/8 glasses per day/9 glasses per day/10 glasses per day/11 glasses per day/12 glasses/day/13glasses per day/14 glasses per day/15 glasses per day/16 glasses per day/17 glasses per day/18 glasses per day/19 glasses per day/20 glasses per day/21 glasses per day/22 glasses per day/23 glasses per day/24 glasses per day/25 glasses per day/26 glasses per day/27 glasses per day/28 glasses per day/29 glasses per day/30 glasses per day

**Bahagian 1: Soal selidik sosio-demografi dan klinikal (Versi Bahasa Malaysia):**

**Kod penyelidikan:**

**Tarikh:**

**Arahan: Dibawah ialah soalan-soalan demografi dan klinikal yang harus dijawab oleh semua peserta kajian. Anda diminta untuk menjawab semua soalan berikut.**

***Sila tanda kotak yang berkenaan berkaitan dengan sama ada anda menggunakan ketum atau tidak.**

Saya menggunakan ketum Saya tidak pernah menggunakan ketum

**(Jika anda menggunakan ketum, sila menjawab soalan-soalan di Bahagian 2 setelah menjawab semua soalan di Bahagian 1)**

**Bahagian A: Butiran sosio-demografi**

(1) Berapakah umur anda?

tahun

(2) Apakah jantina anda?

Lelaki Perempuan

(3) Apakah bangsa anda?

Melayu Bukan Melayu

(4) Apakah status pekerjaan anda?

Bekerja atau kerja sendiri Tidak bekerja

**Bahagian B: Butiran klinikal**

(1) Adakah anda merokok?

Saya merokok Saya tidak merokok

(a) Jika anda merokok, berapakah purata batang hisap yang anda gunakan dalam satu hari?

1-5 batang rokok dalam sehari

6-10 batang rokok dalam sehari

Lebih daripada 10 batang rokok dalam sehari

(2) Adakah anda melakukan aktiviti fizikal yang mencukupi dalam seminggu? (Sila ambil perhatian bahawa: Aktiviti aerobik yang berintensiti sederhana termasuk bermain tenis/badminton (acara bergu), berjalan kaki dengan pantas untuk sekurang-kurangnya 4 km, berbasikal kurang daripada 16 km sejam, menari, berkebun, aerobik dalam air dan sebagainya. Aktiviti aerobik yang berintensiti tinggi termasuk berlari, berbasikal sekurang-kurangnya 16 km sejam, bermain tenis/badminton (acara berseorangan), berenang dalam beberapa pusingan, mendaki bukit, berlompat tali, melakukan kerja rumah yang berat seperti mencangkung tanah, tarian aerobik dan sebagainya).

Aktiviti fizikal yang mencukupi (Saya melakukan aktiviti aerobik berintensiti

sederhana sekurang-kurangnya 150 minit seminggu atau aktiviti aerobik yang

berintensiti tinggi sekurang-kurangnya 75 minit seminggu)

Aktiviti fizikal yang tidak mencukupi (Saya melakukan aktiviti aerobik berintensiti

sederhana kurang daripada 150 minit seminggu atau aktiviti aerobik yang

berintensiti tinggi kurang daripada 75 minit seminggu)

(3) Sila ambil perhatian bahawa salah satu staf kami akan mengukur berat badan dan ketinggian anda untuk mengira indeks jisim badan.

Berat badan = kg

Ketinggian = m

Indeks jisim badan = kg/m^2^

**Bahagian 2. Soal selidik butiran penggunaan ketum (Versi Bahasa Malaysia)**

**Arahan: Soalan-soalan dibawah adalah berkaitan dengan butiran penggunaan ketum bagi mereka yang menggunakan ketum. Sila jawab semua soalan yang berkaitan.**

(1) Berapakah umur anda apabila anda mula menggunakan jus/teh ketum?

Sebelum 18 tahun

18 tahun dan keatas

(2) Berapa tahun anda telah menggunakan jus/teh ketum?

1-5 tahun

Lebih daripada 5 tahun

(3) Dalam purata, berapa kali dalam sehari anda akan meminum jus/teh ketum? Sila bulatkan jawapan anda.

1 kali sehari/2 kali sehari/3 kali sehari/4 kali sehari/5 kali sehari/6 kali sehari/7 kali sehari/8 kali sehari/9 kali sehari/10 kali sehari/11 kali sehari/12 kali sehari/13 kali sehari/14 kali sehari/15 kali sehari/16 kali sehari/17 kali sehari/18 kali sehari/19 kali sehari/20 kali sehari

(4) Dalam purata, anda akan meminum berapa gelas jus/teh ketum dalam sehari? Sila bulatkan jawapan anda.

1 gelas sehari/2 gelas sehari/3 gelas sehari/4 gelas sehari/5 gelas sehari/6 gelas sehari/7 gelas sehari/8 gelas sehari/9 gelas sehari/10 gelas sehari/11 gelas sehari/12 gelas sehari/13 gelas sehari/14 gelas sehari/15 gelas sehari/16 gelas sehari/17 gelas sehari/18 gelas sehari/19 gelas sehari /20 gelas sehari/21 gelas sehari/22 gelas sehari/23 gelas sehari/24 gelas sehari/25 gelas sehari/26 gelas sehari/27 gelas sehari/28 gelas sehari/29 gelas sehari/30 gelas sehari
